# Natural glass alteration under a hyperalkaline condition for about 4000 years

**DOI:** 10.1038/s41598-022-20482-3

**Published:** 2022-09-26

**Authors:** Ryosuke Kikuchi, Tsutomu Sato, Naoki Fujii, Misato Shimbashi, Carlo A. Arcilla

**Affiliations:** 1 Division of Sustainable Resources of Engineering, Faculty of Engineering, Hokkaido University, Sapporo, Hokkaido 060-8628 Japan; 2 Geological Disposal Barrier System R&D Division, Radioactive Waste Management Funding and Research Center, Nichirei Akashicho Bldg.12F, 6-4, Akashicho, Chuo-Ku, Tokyo 104-0044 Japan; 3 Sustainable System Research Laboratory, Central Research Institute of Electric Power Industry, 1646 Abiko, Abiko, Chiba 270-1194 Japan; 4 Department of Science and Technology, Philippine Nuclear Research Institute, Diliman, Quezon City, 1101 The Philippines; 5 National Institute of Geological Sciences, University of the Philippines, Diliman, Quezon City, 1101 The Philippines

**Keywords:** Geochemistry, Mineralogy

## Abstract

Silicate glasses are durable materials in our daily life, but corrosion rate accelerates under alkaline aqueous environment. Such situation has raised concerns, for example, in nuclear waste disposal where vitrified wastes encounter to alkaline leachate from surrounding concrete materials. Here we report volcanic glass example surviving with a hyperalkaline groundwater (pH > 11) and high flow rate for about 4000 years. The tiny glass fragments were extracted from the volcanic ash layer sandwiched between ultramafic sediments using microanalytical techniques. Sharp elemental distributions at the glass surface, where amorphous-like smectite precursors and crystalline smectites coexist, suggest the corrosion by an interface-coupled dissolution–precipitation mechanism rather than inter-diffusion. The corrosion rate was maintained at, the minimum, 2.5 orders of magnitude less than the rate observed for fresh glass, even in the presence of Fe and Mg that might have consumed Si through the silicate precipitation.

## Introduction

Silicate minerals and glasses are ubiquitous materials in the Earth’s surface, and also essential components for industrial and biomedical materials including ceramics, cement, gels and glass. The reactions occurring on the surface of silicate materials with aqueous fluid have been intensively investigated in geosciences because geochemical/biogeochemical weathering govern element cycles in ocean and Earth’s crust^1–3^, as well as in material sciences to enhance technological applications and durability of glass products^4–7^. The long-term interactions with water are particularly important, especially for borosilicate glass, which is used as the host matrix for high level radioactive wastes (HLW), to predict radionuclide retention potential.

Glass corrosion is a complex phenomenon since glass alters across different stages through multiple parallel mechanisms including ionic exchange, hydrolysis of the silica network, gel layer formation and precipitation of secondary phases such as phyllosilicates and zeolites^8–12^. Surface alteration layer forms by diffusion and selective cation exchange, called as “inter-diffusion model"^8,9,13^, and/or by stoichiometric glass dissolution and precipitation of secondary crystalline phases, called as “interface-coupled dissolution–precipitation model”^14,15^. Initial glass alteration is controlled by glass dissolution at the initial rate (Stage I), and then, dissolution rate diminishes to a “residual” rate (Stage II), generally several orders of magnitude lower than the initial rate. Thereafter, a resumption of alteration potentially occurs (Stage III) for certain glass compositions and conditions (e.g., temperature, pH). Past studies showed that hyperalkaline conditions may favor Stage III with rates close to initial rates^16–20^. The return of initial dissolution rates must be one of the worst scenarios for nuclear glass; This can be achieved by the interaction with hyperalkaline plumes from cement materials, which is a ubiquitous component of all geological disposal facilities as backfill/barrier material or as structural support. Thus, the durability of glass under hyperalkaline condition is a key issue.

Since glass alteration behavior cannot be ensured under hyperalkaline conditions beyond a few thousands years, it must be examined using geological or archaeological analogues, as along with various laboratory experiments that asses reaction mechanisms at glass-water interfaces. To date, basaltic glass has been widely investigated, which has reacted under submarine environments with pH ~ 8^21,22^, however, no one has reported natural glass altered by hyperalkaline conditions. This is because there are very few natural environments that offer hyperalkaline fluids on Earth. Studies in saline lakes can provide an opportunity to examine alteration behavior under mildly alkaline conditions (9 < pH < 10)^23^, but lake water rarely exceeds pH ~ 10.5 because of the universal presence of Mg in the natural water leading to the buffering of Mg(OH)_2_ precipitation. With a few exceptions such as deep aquifers of ultramafic rocks (e.g., Oman ophiolite; pH 10–12)^24^ and natural cement analogue (Maqarin Natural Analogue Site in Jordan; pH 12.5–13)^25–27^, groundwater pH rarely reaches 10.5–13 at which Ca(OH)_2_ buffer works, or to even higher. Therefore, it is not surprising that no natural analogue of glass alteration in hyperalkaline environments have been reported to date. In this context, we report a natural glass surviving under hyperalkaline condition (pH > 11) for several thousand years. Although our study is not a complete analogue for a young cement leachate (pH 12.5–13.5)^28^, it corresponds to the leachate from a low alkali cement which has been developed to reduce chemical gradient across repository components^29^, and also the highest pH record for the natural case on glass alteration so far. This study is hoped to provide insights into the mechanisms of glass alteration in hyperalkaline and suggestions that should be verified in future laboratory experiments.

## Results

### Site description

**Figure 1:**
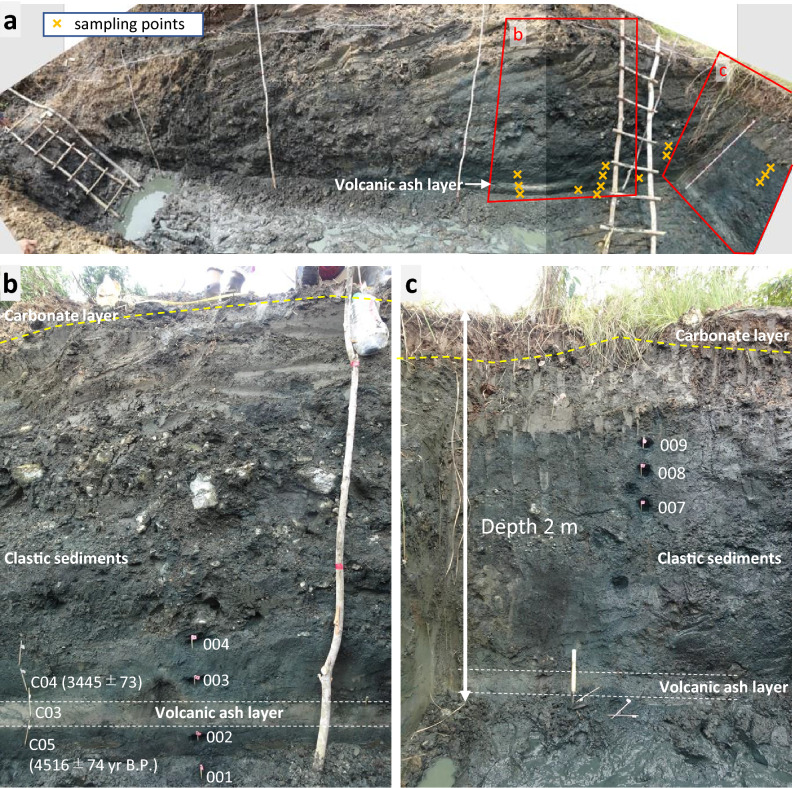
Outcrop photographs of the ultramafic clastic sediment containing a volcanic ash layer: (**a**) Overhead shot photograph of a trench where the crosses denote sampling points. (**b**,**c**) Cross-section of the trench. Solid samples were collected at different depth indicated by small flags. The numbers in parentheses are the ^14^C ages of humin extracted from the clastic sediments (years before present).

The geological setting of Palawan, in the Philippines, is dominated by Palawan Ophiolite, which is composed of the Beaufort Ultramafic Complex, Stavely Gabbro, and Espina Formation^30^. The Beaufort Ultramafic Complex commonly possess serpentinized peridotites and dunites. The study area is located in Narra, in central Palawan (9° 12′ 14ʺ N, 118° 16′ 51ʺ E, ~ 70 m above sea level), where alluvial fan deposits spreading on gentle slopes of this serpentinite basement. Several trench or drill hole samplings were performed in this region to investigate the interaction between the ultramafic sediments and alkaline groundwater^31,32^. One of the trenches in the fan toe shows, a cream-colored volcanic ash layer (~ 20 cm) interbedded between the ultramafic sediments (see Fig. 1). The depositional field was likely a calm waterflow environment, as the ash layer was horizontal without any sedimentary structures that would indicate wave effects. The depositional age of the ash was constrained between 4516 ± 73 and 3445 ± 73 years before present, based on the radioactive carbon (^14^C) age of humin, an alkaline-insoluble organic matter, in the upper- and lower-side sediments bordering the volcanic ash layer^33^. The ^14^C age of the volcanic ash itself could not be determined because of its limited carbon content. The constituent minerals are magnesium hornblende, quartz, feldspar and airborne volcanic glass. This mineral assemblage and the chemical composition of the glass (SiO_2_ ~ 78.3 wt%) indicated rhyolitic magma origin. Although the magma source has not yet been identified because the many volcanic arcs are active from the middle Pleistocene to the Holocene around Palawan^34,35^, the depositional age and the mineral assemblage are consistent with those reported in seafloor core records and with those reported in on-land geological records of the Maraunot eruption period of Pinatubo volcano, 2300–3900 years. B.P.^36,37^.

Table 1 shows the on-site measurements and concentrations of the major cations and anions of alkaline seepage from the bottom of the trench. This seepage is characterized by a high pH (~ 11) and Ca–OH type composition which originates from the interaction of meteoric water with ophiolite^32^. This characteristic is similar to that of leachates from cement degradation, particularly leachates from low-alkali cements. The depositional environment of the trench area is estimated to have once been a brackish environment where alkaline groundwater mixed with seawater;, this then, gradually shifted to the present groundwater-dominated environment^31^. The carbonate layer overlying the ultramafic clastic sediments was formed by mixing of alkaline groundwater with surface fresh water following marine regression. The ^14^C age of calcium carbonate collected from the bottom of the carbonate layer is 2771 ± 73 years B.P^33^, indicating that the clastic sediments beneath the carbonate layer interacted with alkaline groundwater for at least 2800 years ago. The estimated specific discharge of alkaline groundwater was approximately 1.26 m day^−1^^31^ and if we assume hydraulic gradient of 0.035–0.061 (reasonable values for hilly terrains and mountainous area) this gives a hydraulic conductivity at 21–36 m day^−1^, corresponding to the sandy sediments (10–300 m day^−1^)^38^. This groundwater flow is much faster than that observed in deep underground, where groundwater moves several millimeters to meters per year. Thus, a large mass transfer by advection through the ultramafic sediments and volcanic ash layer is expected.

**Table 1:** On-site measurements and concentrations of major cations and anions in ppm (data from previous report^32^).

Aqueous chemical species	Concentrations (ppm)
Temperature (°C)	27.7
pH	11.16
ORP (mV)	− 141
Na^+^	49.1
K^+^	2.02
Mg^2+^	< 0.01
Fe^(2+, 3+)^	0.02
Al^3+^	0.18
Ca^2+^	26.8
Si^4+^	4.27
Cl^−^	28.6
SO_4_^2−^	0.63
HCO_3_^−^	8.9

### Altered glass characteristics

**Figure 2:**
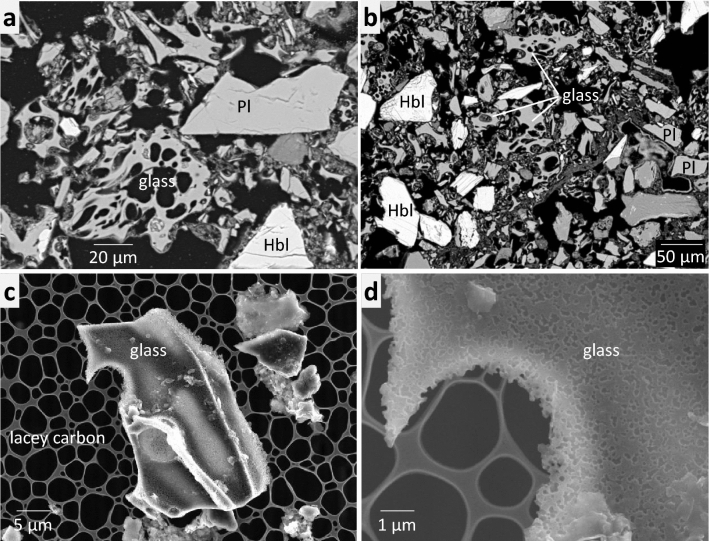
Morphology of volcanic glass. (**a**,**b**) Back-scattered electron images of thin section containing glass. (**c**,**d**) Secondary electron images of glass dispersed on a lacey carbon after ultrasonification. Pl: plagioclase, Hbl: hornblende, Qz: quartz.

The volcanic ash could not be directly examined using bulk analysis because it is intermingled with ultramafic sediments. However, aggregates of volcanic ash can be observed on a microscopic scale. Unlike other constituent minerals, such as plagioclase and hornblende, volcanic glasses have a distinctive bubble-wall shape, and some of the glasses were fragmented (Fig. 2). The glass margins are entirely or partly covered with low dense materials (Fig. 2a,b), corresponding to the so-called “palagonite” texture, which has been recognized a mixture of secondary minerals such as different types of clays, zeolites, hydroxides and oxides^21,39^. Numerous pores appear on the surface of the glass, which were probably caused by chemical attack of alkaline water (Fig. 2d). The chemical composition of the glass is rhyolitic^40^ (78.3 SiO_2_, 13.9 Al_2_O_3_, 3.1 K_2_O, 2.6 Na_2_O, 1.3 CaO, 0.2 MgO in wt%) based on SEM-EDS and a water-free assumption. The chemical composition of the glass was homogeneous between grains and no zoning was observed within the grains.

**Figure 3:**
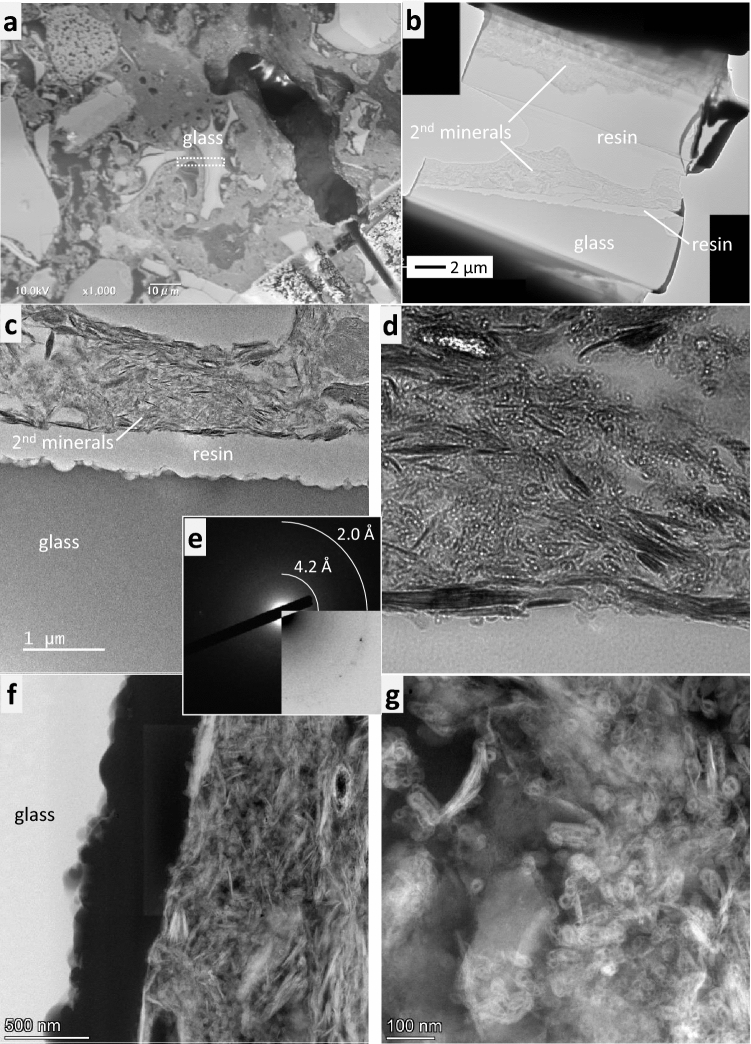
Electron microscope images of altered glass surface. (**a**) The white rectangle denotes micro-sampling position by focused ion beam. (**b**–**d**) Bright-field TEM images showing the glass surface and secondary minerals. (**e**) The selected area electron diffraction pattern obtained from an aggregate of spherical particles; the lower-right side is inversed in contrast. Diffused rings correspond to ~ 4.2 and 2.0 Å. (**f**,**g**) High-angle annular dark-field (HAADF) images.

The glass interface was extracted using a focused ion beam (FIB), and was then observed by (scanning) transmission electron microscopy ((S)TEM) (Fig. 3). The secondary minerals around the glass were found to be a mixture of phyllosilicates and hollow spherical nanoparticles (approximately 20–30 nm in diameter). The phyllosilicate grains were slightly oriented towards the glass surface and adhesion between these secondary minerals and the glass appeared to be weak; the epoxy resin readily penetrated the gaps. While the top surface of the glass was uneven and porous, the inside of the glass, including near the surface, seemed to be dense with no gel-like layer observed (Fig. 3c).

**Figure 4:**
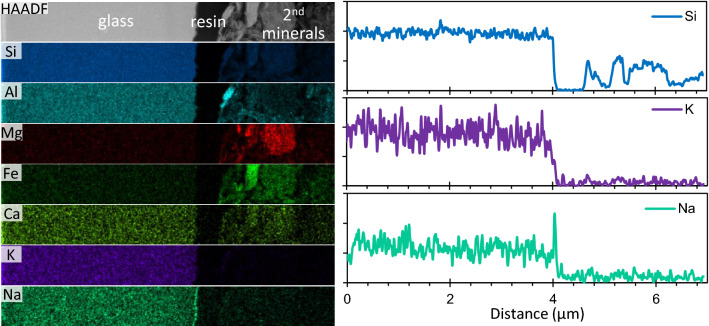
Elemental distributions and profiles across glass-2nd minerals interface. Profiles were obtained horizontally from the left to the right side of the mapping area and were integrated at a width of 100 nm.

Figure 4 depicts the elemental distributions (left) and profiles (right) from the glass to the secondary minerals. The line profile of Si shows a constant intensity inside the glass and a sharp edge at the glass surface without a distinguishable inter-diffusional layer. K and Na have a slightly gentler edge compared to that of Si, and their transition zones from the interface between the glass surface and resin toward the inside of the glass are less than approximately 400 nm in width. This suggests corrosion by stoichiometric dissolution at the glass surface, not by development of an inter-diffusional layer, although the diffusion of water molecules into the glass could not be ruled out.

## Discussion

The elements released from the glass surface by stoichiometric dissolution were considered to have reprecipitated, depending on the degree of saturation of the interfacial solution. The texture containing spherical, amorphous-like nanoparticles and more crystalline phyllosilicates was consistent with the characteristics of palagonite, as has been previously reported^39,41,42^. The spherical particles had an Al/Si ratio of ~ 0.74, suggesting that they had a 2:1 type clay mineral structure. Exfoliation of the 2:1 layer may form a hollow spherical morphology. The phyllosilicate grains surrounding the spherical particles have different compositions for individual grains; Al-rich high-charge smectite, Mg-saponite, Fe-saponite or nontronite (see Supplementary Fig. S2 and Table S2 online). It has been suggested that spherical particles were precursors, which then transformed into phyllosilicates with crystal growth^41^. While spherical particles could be formed from Si and Al released due to glass dissolution, there was insufficient Fe and Mg in the glass to form saponites and/or nontronite. Thus, the supply of Fe and Mg from the surrounding ultramafic sediments could have resulted in their formation. Unlike the dissolution tests under alkaline conditions^43,44^ and the natural basaltic glass alteration^21^, there was no sign of zeolite formation. This is supported by saturation index of the seepage, showing that zeolite phases such as analcime and merlinoite are undersaturated while saponites are oversaturated (see Supplementary Table S3).

**Figure 5:**
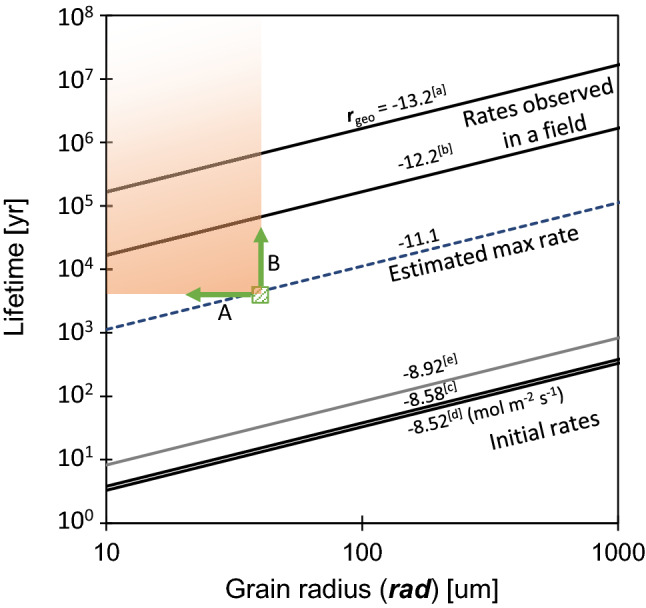
Lifetime of spherical glass grain at a given dissolution rate. The solid lines represent the lifetime of spherical glass as function of the grain radius (***rad***) and dissolution rate. The numbers above the lines denote the dissolution rates log ***r***_**geo**_ (in mol m^−2^ s^−1^). Rates are cited from the following: rhyolite dissolution rates under near neutral pH at field, assuming a roughness factor of[a] 10 and [b] 100, respectively^48^. Rates were determined by experiments conducted at pH 10.6 at 25 °C^46^ [c], as well as at pH 11.2 at 28 °C^47^ [d]. The dissolution rate at pH 9.5, 28 °^47^ [e] is also shown as a gray line. In this study, the values for [c] were converted to match the volcanic glass composition, and the values for [d] and [e] were interpolated from the rates at different pH and temperatures. The broken line across the green square denotes the lifetime estimated at the maximum dissolution rate. Arrows A and B indicate constraints on grain radius and lifetime (see text for details); more realistic radius and lifetime are plotted in the upper left area of the square (shaded orange).

There are two possibilities for the role of the secondary phases. One is that the precipitation of these new phases reduced the saturation state of the interfacial solution, leading to enhanced glass dissolution. The second is that the secondary phases covered the glass’s surface and played a role in obstructed water flow and limited glass dissolution. Did the formation of secondary phases maintain a high dissolution rate, or did they act as a protective coating in the present case? To answer this question, we conducted simple calculations. Although various dissolution models have been proposed, we used the simplest dissolution model for spherical grains^45^, to estimate the time ***t*** required to completely dissolve, as shown in the following equation:

1 $${\varvec{t}}_{{{\text{lifetime}}}} = \left( {{\varvec{rad}}/{\varvec{V}}_{{\text{m}}} {\varvec{r}}_{{{\mathbf{geo}}}} } \right)$$

where ***rad*** denotes the grain radius, ***V***_m_ is the molar volume where a mole of glass is assumed to contain one Si atom, and ***r***_**geo**_ refers to the dissolution rate normalized by the geometric surface area. Using Eq. (1), the lifetime of spherical glass can be predicted for different dissolution rates (Fig. 5). To assume that an initial dissolution rate is maintained consistently, the rates determined by laboratory tests under far from equilibrium were cited^46,47^. Both have similar values, and, for example, a glass sphere with a 100 μm radius will be completely dissolved within several tens of years. The dissolution rates found in geological samples are thought to be lower than the initial rates, for example, rhyolitic glass dissolution rates determined through field experiments, at a neutral, gives the glass a longer lifetime^48^. In the present case, although we could not exactly know the original size of the volcanic glass, it was considered to have a radius of ~ 40 μm at maximum based on the grain size of the surrounding crystalline minerals, such as hornblende (see Supplementary Fig. S3). This estimated grain size of the glass and its depositional age (i.e., 3445–4516 years) provide the dissolution rate of ~ 10^−11.1^ (in mol m^−2^ s^−1^), as plotted as the green square marker in Fig. 5. Owing to the bubble-wall shape of the glass, the actual specific surface area is expected to be much larger than that of the spherical shape. Thus, it is clear that calculations assuming a grain size < 40 μm is more practical and then, the marker should plot to the left side of its current position (as indicated by arrow A in Fig. 5). Since the glass has not yet completely dissolved, it is obvious that its actual lifetime is longer than its depositional age. Depending on how long the glass will survive in the future, the marker should plot to the upper side of its current position (as indicated by arrow B in Fig. 5). These two constraints indicate that the estimated dissolution rate of 10^−11.1^ is a maximum value and that the actual rate must be slower than this. Although this is a rough estimation, the dissolution rate is at least 2.5 orders of magnitude slower than the initial dissolution rate in the alkaline solution. Similarly, this estimated rate is at least 2.2 orders of magnitude slower than the initial dissolution rate at pH 9.5, assuming an initial depositional environment where seawater and alkaline groundwater are mixed^31^. Since there is no development of a thick inter-diffusional layer on the glass surface, a retardation effect on dissolution due to diffusion inside the glass is not expected. Rate-limiting due to the saturation of dissolved silica in the mainstream of the groundwater is unexpected as well, since high groundwater velocity keeps silica concentration low level as that of ordinary groundwater even if Si is supplied by glass dissolution^49^. Therefore, it is reasonable to assume that the secondary minerals acted as a protective film over the glass rather than the accelerated dissolution of the glass by the consuming dissolved silica in the interfacial solution.

Note that our observations are probably not easily transferable to other systems with different glass compositions, temperatures, water flow rates and time-scales. Despite this, our findings emphasize the importance of secondary minerals in long-term glass alteration processes. In hyperalkaline environments, zeolite formation has been reported as a secondary mineral around glass^16,43,44^, but in the presence of Mg or Fe, phyllosilicates may form preferentially. In the geological repository system, groundwater is expected to contain Mg from host rocks and seawater intrusion in the coastal area, while Fe is released by the corrosion of metallic materials in the multi-barrier repository concept^50^. In short-term alterations, the consumption of Si in the glass is likely to be accelerated by the formation of secondary products bearing Mg and Fe^51^. Fe is generally considered to accelerate glass alteration by forming secondary products and creating less-protective gel layer^52^. For Mg, the formation of trioctahedral smectites or Mg incorporated into the gel layer on the glass surface have been reported^53,54^. In the long-term alteration, on the other hand, the role of secondary products may become more complex as alteration progresses. While silicate formation consumes Si, these secondary products limit mass transport between the main flow path of groundwater and the pristine glass and thus do not prevent a significant decrease in the glass alteration rate. The in-situ corrosion tests and the reactive transport modeling that simulate glass corrosion under geological repository conditions have confirmed the complex role of Mg- and Fe-silicates^55^. This study supports that this is valid even under hyperalkaline conditions and for very long periods of time. The formation of secondary products, especially swelling smectites, could fill the pore space and inhibit further glass alteration by limiting water accessibility even in high flow rate systems. The effect of pore clogging is expected to be more dominant in the geological media where the reaction process is controlled by diffusion owing to slower advection.

Although some of the secondary minerals are considered to be formed during early glass alteration, where mixing with groundwater and seawater lowered the pH to about 9.5^31^, the ^14^C age of the carbonate layer overlying clastic sediments indicates that alkaline groundwater with a pH of 11.2 was the dominant from ~ 2800 years ago to the present. The time scale in this study is much longer than that of laboratory experiments, and yet, is relatively short period from a geological perspective. Studies on this timescale are important in natural environments where human activity is involved. In the context of geological disposal, water-soluble nuclides in vitrified wastes such as ^137^Cs and ^90^Sr, which account for most of the initial radioactivity, will decay in the first several hundreds to thousand years. Thus, it is desirable that retention potentials be maintained during the first thousand years, even if vitrified wastes encounter groundwater and/or cement leachates. In addition to high-level radioactive wastes, glassy radiocesium (^137^Cs and ^134^Cs)-bearing microparticles that were released by nuclear power plant accidents such as Fukushima also require safe isolation from human activity zones^56^. Glass alteration in the first few 100 years for radioactive decay is a key issue. Glass durability for several tens to hundreds of years should be predicted in the field of radioactive waste management, through a comparison of laboratory experiments, geochemical modeling and archaeological/geological analogues in these waste management systems, as well as in other fields of civil engineering using glass fiber reinforced cements^57^, and CO_2_ sequestration via carbonation of basaltic glass^58^.

The unique geological environments of Narra studied here provided insights into long-term glass alteration under hyperalkaline conditions. Although bulk analysis, such as BET surface area measurement, cannot be applied, recent advanced microanalytical techniques enable us to examine not only massive basaltic glasses formed on the sea floor but also tiny volcanic glasses formed rapidly in the atmosphere. This suggests the potential for investigating long-term glass alteration by studying volcanic glass in other volcanically active regions. Our knowledge of the long-term durability of glass will be more robust if natural examples can be collected in various aqueous environments, similar to what we do in routine laboratory works running under various conditions.

## Methods

The sediment containing volcanic ash was embedded in epoxy resin and dry-polished to create a flat surface. Polished sections were observed using a scanning electron microscope (SEM) with an energy-dispersive spectrometer (JSM IT-200, JEOL). An electron transparent foil (approximately 150 nm) for (S)TEM observations was prepared by focused ion beam (FIB-SEM, JIB4600F, JEOL). This foil was then analyzed using a JEM-2100F (JEOL) at an accelerate voltage of 200 kV, and Titan G2 (FEI) at 60 kV. For further details, see Supplementary Information.

## Supplementary Information

Supplementary Information 1.

Supplementary Information 2.

## Data Availability

The authors confirm that the data supporting the findings of this study are available within the article and its supplementary materials.
