# Supplementary material for: Natural glass alteration under a hyperalkaline condition for about 4000 years
Source: Sci Rep. 2022 Sep 26;12:16012. doi: 10.1038/s41598-022-20482-3 (PMC9512812; doi:10.1038/s41598-022-20482-3)
Supplement: Supplementary file 1 — Supplementary Information 1. [file 41598_2022_20482_MOESM1_ESM.pdf]

Supplementary information

Natural glass alteration under a hyperalkaline condition for about 4000 years

Ryosuke Kikuchi*, Tsutomu Sato, Naoki Fujii, Misato Shimbashi, Carlo A.Arcilla

1. Sample preparation

Several centimeter-sized blocks of sediment containing volcanic ash were embedded in epoxy resin and hardened at room temperature. Polishing was performed using a complete dry procedure without using any use of water or oil. In addition to thin-sections, dispersed specimens were also prepared. A small amount of sediment block was dispersed in ethanol, and then the suspension after ultrasonification was dropped on the membrane of a lacey-carbon coated 200 mesh copper grids. A carbon coating was applied to both thin sections and dispersed specimens to give them electron conductivity.

2. Electron microscopy

Scanning electron microscopy with energy dispersive spectrometry (JSM IT-200, JEOL) was performed at an accelerating voltage of 15 kV. The chemical composition of the volcanic glass, based on SEM-EDS and the assumption of water free is shown in Table S1; 10 point-analyses were averaged.

Table S1. Chemical composition of the glass

	wt%	S.D.%
Na_2O	2.62	0.11
MgO	0.19	0.10
Al_2O_3	13.86	0.14
SiO_2	78.29	0.53
K_2O	3.14	0.22
CaO	1.33	0.20
total	100	

An electron transparent foil (approximately 150 nm) for (S)TEM observations was prepared by focused ion beam (FIB-SEM, JIB4600F, JEOL). The FIB instrument was operated at 30 kV to make the specimen thickness <150 nm, and then at 10 kV during the final thinning process to avoid amorphous artefact formation on the foil surface and beam damage. The foil was analyzed using a JEM-2100F (JEOL) at an accelerating voltage of 200 kV. The same foil was also analyzed using Titan^3 G2 60-300 (FEI) with a monochromator and double spherical aberration correctors. An acceleration

voltage of 60 kV and probe current of 100 pA were used.

The entire interface between the glass and secondary minerals is shown in Fig. S1. Line profiles of the constituent elements across the interface are also displayed.

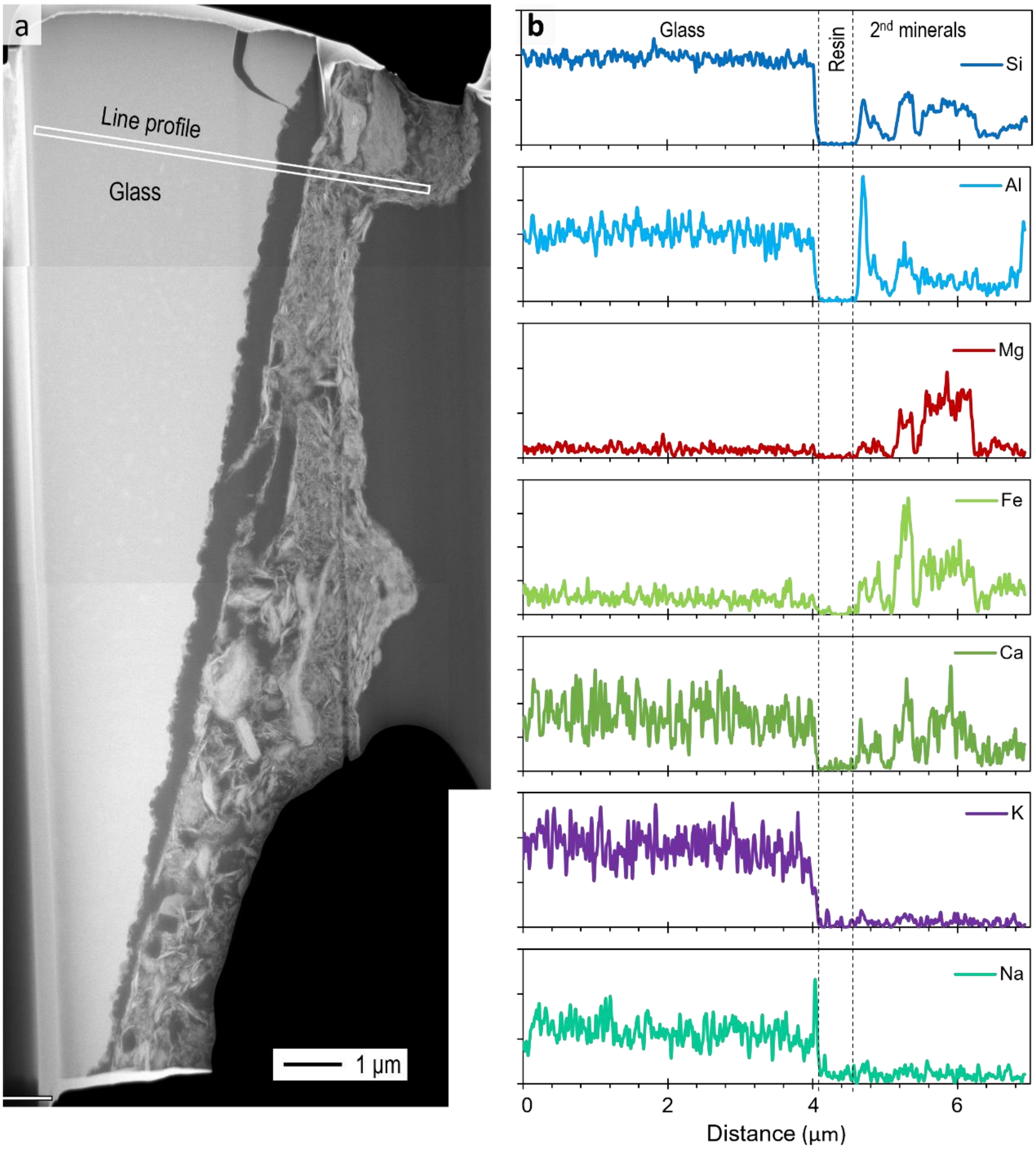

Fig. S1. HAADF-STEM image (a) and elemental profiles across glass-2nd minerals interface (b). Line profiles was taken from the left to the right side of the white rectangle with integrated at a width of 100 nm.

The composition of the secondary minerals was examined using STEM-EDS (Fig. S2). This illustrates that spherical nanoparticles and more crystalline smectites aggregate together. The chemical

compositions are also shown in Fig.S2. Areas #2, 3 and 4 represent Mg-, Fe-, and Al-rich areas, respectively. Their structural formula on the basis of $O_{10}(OH)_2$ were calculated using the assumption of Fe^{2+} or Fe^{3+} (Table S2). The Mg-rich area (i.e. #2) is considered to be saponite, although its octahedral sheet has comparable amounts of Al and Mg, and slightly less Fe. The Fe-rich area was interpreted as ferrian-saponite or nontronite. The Al-rich area can be interpreted as high-charge smectite or dioctahedral vermiculite, based on their isomorphic substitution of tetrahedral sheet. Considering the formation environment of dioctahedral vermiculite in the literature, its formation in Narra is not likely due to the absence of micaceous precursors; it is thus more plausible to consider it as a high-charge smectite. To compensate for the negative charge of the tetrahedral sheet, part of Fe and Mg are suspected to be placed at the interlayer in addition to Ca. The similarity of the composition of Areas #1 and #4 probably indicates that the low-crystalline, spherical nanoparticles correspond to the precursor of Al-rich high-charge smectite.

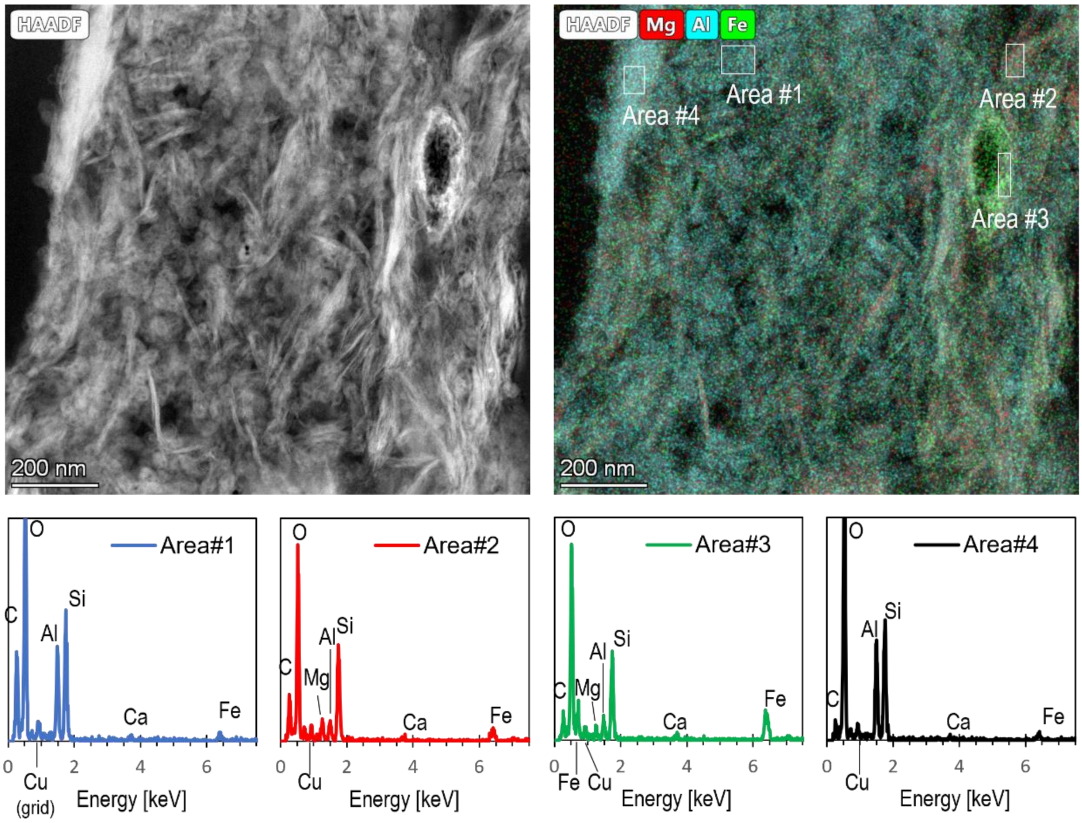

Fig. S2. Elemental distributions of secondary minerals.

The two images in the upper side are HAADF-STEM image and superimposed images including Mg, Al and Fe distributions. The lower side shows four STEM-EDS spectra obtained from different areas (i.e., Areas #1- #4 on the upper-right side).

Table S2. Cationic distributions based on $O_{10}(OH)_2$

	Assumption of Fe^{3+}					Assumption of Fe^{2+}			
	#1	#2	#3	#4		#1	#2	#3	#4
Si	3.37	4.03	3.70	3.21	Si	3.34	3.92	3.45	3.18
$^{[IV]}Al$	0.63	0.00	0.30	0.79	$^{[IV]}Al$	0.66	0.08	0.55	0.82
Σtet	4.00	4.03	4.00	4.00	Σtet	4.00	4.00	4.00	4.00
$^{[VI]}Al$	1.87	0.87	0.57	1.94	$^{[VI]}Al$	1.81	0.76	0.26	1.88
Fe^{3+}	0.21	0.61	1.62	0.22	Fe^{2+}	0.21	0.59	1.51	0.22
Mg	0.20	0.87	0.44	0.15	Mg	0.20	0.85	0.41	0.15
Σoct	2.28	2.35	2.64	2.31	Σoct	2.22	2.20	2.18	2.25
Ca	0.11	0.17	0.22	0.11	Ca	0.10	0.16	0.21	0.11

3. Mineral saturation index

The water quality data of alkaline seepage from the bottom of the trench were obtained from the previous report of on-site measurements and concentrations of major cations and anions[1]. Chemical species activities and mineral saturation index was calculated using the Geochemist's Workbench with the thermodynamic database, "Thermoddem_V1.10_1Dec2020" provided by the Bureau de Resherches Géologiques et Minieres (BRGM). Ca-, Mg-, and Fe- bearing mineral species among the zeolite and smectite groups were calculated (Table S3).

Table S3. Mineral saturation index

Mineral names*	Chemical composition	Saturation index (log Q/K)
Analcime	$Na_{0.99}Al_{0.99}Si_{2.01}O_6{:}H_2O$	-3.889
Merlinoite(K)	$K_{1.04}Al_{1.04}Si_{1.96}O_6 \cdot 1.69H_2O$	-7.307
Merlinoite(Na)	$Na_{1.04}Al_{1.04}Si_{1.96}O_6 \cdot 2.27H_2O$	-6.512
Chabazite	$Ca(Al_2Si_4)O_{12} \cdot 6H_2O$	-3.463

Phillipsite(Ca)	$Ca_{0.5}AlSi_3O_8 \cdot 3H_2O$	-4.052
Saponite(Ca)	$Ca_{0.17}Mg_3Al_{0.34}Si_{3.66}O_{10}(OH)_2$	4.258
Saponite(FeCa)	$Ca_{0.17}Mg_2FeAl_{0.34}Si_{3.66}O_{10}(OH)_2$	-0.753
Saponite(FeMg)	$Mg_{0.17}Mg_2FeAl_{0.34}Si_{3.66}O_{10}(OH)_2$	-0.764
Saponite(Mg)	$Mg_{0.17}Mg_3Al_{0.34}Si_{3.66}O_{10}(OH)_2$	4.245
Saponite(SapCa)	$Na_{0.394}K_{0.021}Ca_{0.038}(Mg_{2.949}Fe_{0.055})(Si_{3.569}Al_{0.397})O_{10}(OH)_2$	3.459
Hsaponite(Ca)	$Ca_{0.17}Mg_3Al_{0.34}Si_{3.66}O_{10}(OH)_2 \cdot 4.799H_2O$	7.132
Hsaponite(FeCa)	$Ca_{0.17}Mg_2FeAl_{0.34}Si_{3.66}O_{10}(OH)_2 \cdot 4.799H_2O$	2.122
Hsaponite(FeMg)	$Mg_{0.17}Mg_2FeAl_{0.34}Si_{3.66}O_{10}(OH)_2 \cdot 5.039H_2O$	2.324
Hsaponite(Mg)	$Mg_{3.17}Al_{0.34}Si_{3.66}O_{10}(OH)_2 \cdot 5.039H_2O$	7.333
Nontronite(Ca)	$Ca_{0.17}Fe^{3+}_{1.67}Al_{0.67}Si_{3.66}O_{10}(OH)_2$	-2.494
Nontronite(Mg)	$Mg_{0.17}Fe_{1.67}Al_{0.67}Si_{3.66}O_{10}(OH)_2$	-2.507
Nontronite(Nau2)	$Na_{0.247}K_{0.02}(Fe^{3+}_{1.688}Al_{0.276}Mg_{0.068})(Si_{3.458}Al_{0.542})O_{10}(OH)_2$	-1.873
Hnontronite(Ca)	$Ca_{0.17}Fe^{3+}_{1.67}Al_{0.67}Si_{3.66}O_{10}(OH)_2 \cdot 4.24H_2O$	-0.675
Hnontronite(Mg)	$Mg_{0.17}Fe_{1.67}Al_{0.67}Si_{3.66}O_{10}(OH)_2 \cdot 4.098H_2O$	-0.505
Montmorillonite(HcCa)	$Ca_{0.3}Mg_{0.6}Al_{1.4}Si_4O_{10}(OH)_2$	-3.809
Montmorillonite(HcMg)	$Mg_{0.3}Mg_{0.6}Al_{1.4}Si_4O_{10}(OH)_2$	-3.888
Montmorillonite(MgCa)	$Ca_{0.17}Mg_{0.34}Al_{1.66}Si_4O_{10}(OH)_2$	-6.066
Hmontmorillonite(HcCa)	$Ca_{0.3}Mg_{0.6}Al_{1.4}Si_4O_{10}(OH)_2 \cdot 4.288H_2O$	-1.291
Hmontmorillonite(HcMg)	$Mg_{0.3}Mg_{0.6}Al_{1.4}Si_4O_{10}(OH)_2 \cdot 5.129H_2O$	-0.715
Hmontmorillonite(MgCa)	$Ca_{0.17}Mg_{0.34}Al_{1.66}Si_4O_{10}(OH)_2 \cdot 4.265H_2O$	-3.751

*Names registered in thermodynamic database, “Thermoddem_V1.10_1Dec2020”.

4. Estimation of the glass dissolution rate

4.1. Calculating a spherical glass lifetime

To estimate the lifetime of natural glass, the following equation for spherical grain dissolution[2]:

$$t_{\text{lifetime}} = \left(rad/V_{\text{m}}\, r_{\text{geo}}\right) \quad (1)$$

where ***rad*** denotes the grain radius, $\boldsymbol{V}_m$ is the molar volume where a mole of glass is assumed to contain one Si atom and $\boldsymbol{r}_{geo}$ refers to the dissolution rate normalized by the geometric surface area. Based on the chemical composition of the glass (Table S1), its structural formula was $Si_1\ Na_{0.06}\ Mg_{0.003}\ Al_{0.212}\ K_{0.05}\ Ca_{0.02}\ O_{2.39}$ (with molecular weight: 76.26 g mol^{-1}). Since we cannot accurately measure the density of the glass, we assumed 2.4 g cm^{-3} to be as a reasonable value for rhyolitic glass based on the literature[3], and then molar volume $\boldsymbol{V}_m$ was ~31.78 cm^3 mol^{-1}. Thus, given a certain dissolution rate $\boldsymbol{r}_{geo}$, the equation $\boldsymbol{rad}$-$\boldsymbol{t}_{lifetime}$ can be obtained.

4.2. Dissolution rates in literature

For the dissolution rate (in g m^{-2} s^{-1} or mol m^2 s^{-1}) in the literature, there are two ways of using surface area for normalization: one provides a geometric surface area ($\boldsymbol{S}_{geo}$) and the other provides a surface area determined by the Brunauer-Emmett-Teller (BET) method ($\boldsymbol{S}_{BET}$). The standard test methods for glass corrosion often use $\boldsymbol{S}_{geo}$ based on a shrinking core model that uses the initial grain sizes and mass change during the corrosion tests[4]. Since the BET measurement was hardly applied to the glass in this study, we adopted the dissolution rates normalized by $\boldsymbol{S}_{geo}$ rather than, $\boldsymbol{S}_{BET}$.

The dissolution rate for rhyolitic glass at pH 10.6at 25˚C[3] was cited. Based on their proposed equation for the dependence of rates on the SiO_2 concentration, the dissolution rates at 78.3 SiO_2 in wt% was calculated to suit the glass in this study. Note that the pH and temperature deviated slightly from the conditions in this study. Data from another dissolution tests[5] was also cited for comparison. From their data showing dissolution rates versus pH at different temperature (Fig.1 in their report[5]), the dissolution rate at pH 11.2, 28˚C and pH 9.5, 28˚C were interpolated and found to be ~ $10^{-1.7}$ and $10^{-2.1}$ g m^{-2} d^{-1}, respectively. The rates were then converted to ~ $10^{-8.52}$ and $10^{-8.92}$ mol m^{-2} s^{-1} respectively, using the molecular weight of the glass in this study. In the corrosion test conducted in the above[5], they used EWG-C glass (i.e., 42.96 SiO_2 in wt%) and thus, one might question whether it can be used as a dissolution rate for rhyolitic glass; however, they conclude that the effect of chemical compositions on the dissolution rate is smaller than the dependence on temperature and pH. Therefore, it is probably more important to match the pH and temperature with those in the present study, and we adopted their data.

Compared to the experimental works, few literatures have estimated the dissolution rates of rhyolitic glass in the natural environment. We have cited the dissolution rates under near neutral pH in this paper[6]. They normalized the dissolution rate by $\boldsymbol{S}_{BET}$, and $\boldsymbol{S}_{geo}$ is not determined because of the bubbled shapes. Considering that the surface roughness ($\boldsymbol{S}_{BET}/\boldsymbol{S}_{geo}$) of rhyolitic glass was reported to be 13 – 94[3], we assumed that the rhyolitic glass roughness lies between 10 and 100. Thus, a rate one to two orders of magnitude higher than that reported[6] would be the dissolution rate assuming a geometric surface area. Figure 5 shows two lines at the upper and lower limits.

4.3. Estimating the original glass size

In this study, the initial grain size of glass was estimated from the current grain size of crystalline minerals, assuming that there was no significant size change due to the dissolution of crystalline materials. Although Crystalline minerals such as hornblende are thought to be partially dissolved by the reaction of hyperalkaline water, the dissolution rate of crystalline feldspar is approximately three orders of magnitude slower than that of glass[7]. Thus, dissolution of crystalline minerals is expected to be much slower than that of glass. Judging from the grain boundaries of crystalline minerals without a distinguishable alteration layer and the well-preserved bubble-wall shape of the glass, significant dissolution of the crystalline grains was unlikely. Therefore, changes in size due to the dissolution of crystalline minerals is considered negligible, at least for the spatial resolution in the SEM observations.

The grain-size estimation procedure is as follows. The grain size of the constituent minerals of volcanic ash can be visualized using SEM back-scattered electron images at low magnification (Fig. S3). Since the contrast is distinctly higher for hornblende than for glass, feldspar, and quartz, hornblende particles can be easily extracted by setting a threshold on the image contrast. The area of each particle was measured using the particle analysis function in ImageJ. Note that the sample includes not only hornblende but also fine particles with bright contrast (mainly Fe or Ni oxides in the serpentinite lithic). Therefore, particles with an area of less than 10 μm^2 were excluded from the following calculation to remove these fine particles. Then, the radius of each grain was calculated assuming that each grain had a circular shape. The maximum radius of the measured hornblende particles ($n = 600$) was 37.36 μm, and the median was 9.7 μm. The entire list of the analyzed particles can be found in the Supplementary data file (Supplementary Table4). By adopting the maximum radius as a conservative estimation for the dissolution rate, we concluded that the maximum size of the initial glass would be approximately 40 μm.

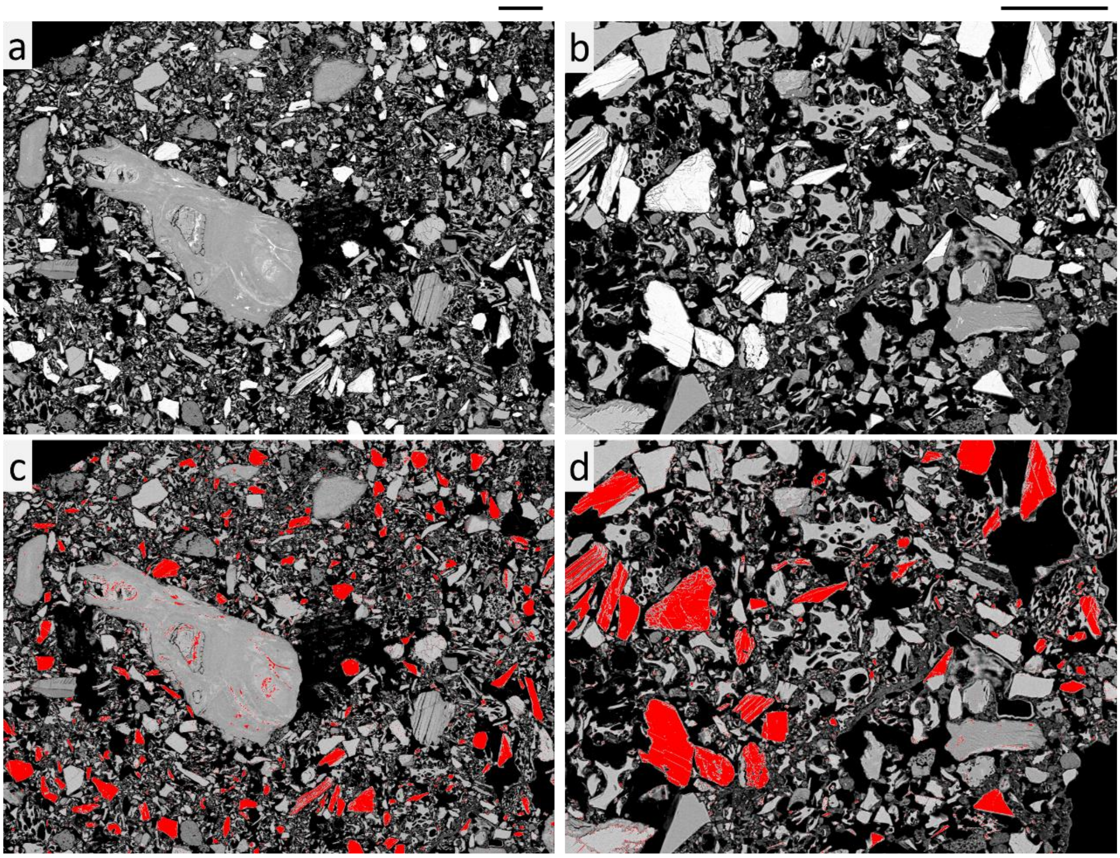

Fig. S3. Particle size analysis for hornblende grains

(a, b) Back-scattered electron images. The largest grain from the center to the upper-left side in (a) is serpentinite lithic. (c, d) Red represents the hornblende grains extracted using a brightness threshold. Scale bars are 100 μm for each image.

References in Supplementary Information

1. Shimbashi, M., Sato, T., Yamakawa, M., Fujii, N. & Otake, T. Formation of Fe-and Mg-rich smectite under hyperalkaline conditions at narra in Palawan, the Philippines. *Minerals* **8**, 1–16 (2018).
2. Lasaga, A. C. *Kinetic theory in Earth Sciences*. (Princeton University Press, 1998).
3. Wolef-Boenisch, D., Gislason, S. R., Oelkers, E. H. & Putnis, C. V. The dissolution rates of natural glasses as a function of their composition at pH 4 and 10.6, and temperatures from 25 to 74°C. *Geochim. Cosmochim. Acta* **68**, 4843–4858 (2004).
4. Thorpe, C. L. *et al.* Forty years of durability assessment of nuclear waste glass by standard methods. *npj Mater. Degrad.* **5**, (2021).
5. Vienna, J. D., Neeway, J. J., Ryan, J. V. & Kerisit, S. N. Impacts of glass composition, pH, and temperature on glass forward dissolution rate. *npj Mater. Degrad.* **2**, 1–12 (2018).

6. Yokoyama, T. & Banfield, J. F. Direct determinations of the rates of rhyolite dissolution and clay formation over 52,000 years and comparison with laboratory measurements. *Geochim. Cosmochim. Acta* **66**, 2665–2681 (2002).

7. Bourcier, W. L. *Affinity functions for modeling glass dissolution rates (No. UCRL-JC-131186). Atomic Energy Commission-Valee due Rhone Summer Workshop: Glass: Scientific Research for High Performance Contaminant Mejannes-le-Clap, France* (1998).
